# AWRAMBA COMMUNITY-BASED CARE FOR ELDERS LACKING FAMILY SUPPORT IN ETHIOPIA

**DOI:** 10.1093/geroni/igz038.571

**Published:** 2019-11-08

**Authors:** Margaret E Adamek, Setegn Ali

**Affiliations:** 1 Indiana University School of Social Work, Indianapolis, Indiana, United States; 2 University of Gondar, Gondar, Ethiopia, Ethiopia

## Abstract

In rural Ethiopia, land fragmentation, poverty, rural-urban migration, and the expansion of market forces are negatively impacting family support for older persons. This study explored an innovative community-based support system that uses its own wealth redistribution mechanism to support congregate residential care for older persons. The Awramba Community in northwest Ethiopia has different social norms and values that make it unique from surrounding communities. To explore this new approach to meeting the needs of rural elders, primary data were obtained from in-depth interviews with 8 elders who reside in the older adult center, 3 focus group discussions, and personal observations. In addition, key informant interviews were conducted with a full-time caregiver, two community leaders, and members of the Older Persons’ Support Committee. Interviews explored the types of services provided to older persons, the interactions of older residents within the community, and the benefits of congregate living. Community members (N=403) must agree to 4 guiding principles relating to gender equality, respecting the rights of children, discouraging dishonesty, lying and stealing, and helping the less fortunate, ill and older persons. The Older Person’s Support Committee is responsible for coordinating the provision of care for the older persons who live in the congregate center. Residing in a common residential home benefited the elders in multiple ways, including opportunities for socialization with peers and access to a full-time caregiver. As traditional family support wanes and the population of elders increases in developing nations, such alternative support strategies are critically needed.

